# The Role of Dopamine and Its Dysfunction as a Consequence of Oxidative Stress

**DOI:** 10.1155/2016/9730467

**Published:** 2015-12-06

**Authors:** Hugo Juárez Olguín, David Calderón Guzmán, Ernestina Hernández García, Gerardo Barragán Mejía

**Affiliations:** ^1^Laboratorio de Farmacología, Instituto Nacional de Pediatría (INP), 04530 Ciudad de México, DF, Mexico; ^2^Departamento de Farmacología, Facultad de Medicina, Universidad Nacional Autónoma de Mexico, 04130 Ciudad de México, DF, Mexico; ^3^Laboratorio de Neuroquimica, INP, 04530 Ciudad de México, DF, Mexico

## Abstract

Dopamine is a neurotransmitter that is produced in the substantia nigra, ventral tegmental area, and hypothalamus of the brain. Dysfunction of the dopamine system has been implicated in different nervous system diseases. The level of dopamine transmission increases in response to any type of reward and by a large number of strongly additive drugs. The role of dopamine dysfunction as a consequence of oxidative stress is involved in health and disease. Introduce new potential targets for the development of therapeutic interventions based on antioxidant compounds. The present review focuses on the therapeutic potential of antioxidant compounds as a coadjuvant treatment to conventional neurological disorders is discussed.

## 1. Introduction to Dopamine

Dopamine (DA) plays a vital role in reward and movement regulation in the brain. In the reward pathway, the production of DA takes place in the ventral tegmental area (VTA), in nerve cell bodies. From there, it is released into the nucleus accumbens and prefrontal cortex. In vivo, the concentration of DA in the VTA is 4.8 ± 1.5 nM, while in red nucleus, it is 0.5 ± 1.5 nM [1]. The pathway for motor functions is different. In this pathway, the substantia nigra cell bodies are responsible for the production and discharge of DA into the striatum. DA plays multiple functions in the brain. Calabresi et al. reported the role of DA in the modulation of behavior and cognition; voluntary movement; motivation; punishment and reward; inhibition of prolactin production; sleep; dreaming; mood; attention; working memory; and learning [2].

**Figure 1:**
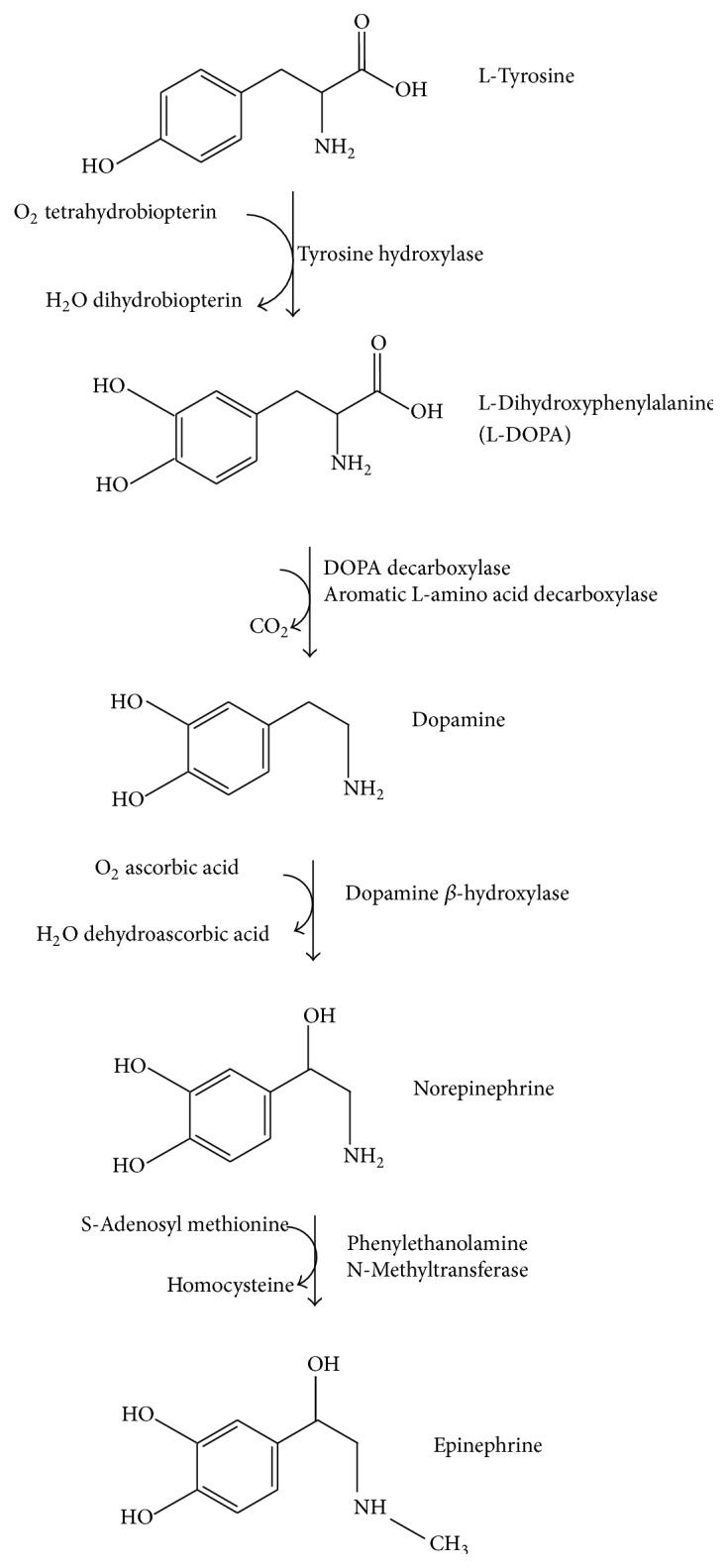
Catecholamine biosynthesis.

DA can be a precursor in the biosynthesis of other related catecholamines such as norepinephrine and epinephrine (Figure 1). Norepinephrine is synthesized from DA by the catalytic action of DA *β*-hydroxylase in the presence of L-ascorbic acid and molecular oxygen (O_2_). Norepinephrine then acted upon by the enzyme phenylethanolamine* N*-methyltransferase with* S*-adenosyl-L-methionine (SAMe) as a cofactor to produce epinephrine.

The biosynthesis of DA and other catecholamines can be limited by the action of enzyme tyrosine hydroxylase (TH) [3]; therefore, regulatory mechanisms of TH could be promising for improving gene therapy approaches and other treatment modalities [4]. After the synthesis of DA, it is incorporated into synaptic vesicles by the action of vesicular monoamine transporter 2 (VMAT_2_), where it is stored. DA is discharged by exocytosis into the cell membrane and dumped into the synapse.

## 2. Dopamine Receptors

In the synapse, DA binds to either postsynaptic or presynaptic DA receptors or both. This bond, regardless of the receptor, generates an electric potential in the presynaptic cell [5]. In the case of postsynaptic DA receptors, the signal is propagated to the postsynaptic neuron, while, in the case of presynaptic DA receptors, the signal can either excite the presynaptic cell or inhibit it. Presynaptic receptors with an inhibitory potential, also known as autoreceptors, inhibit the synthesis and release of neurotransmitters and thus function to maintain normal levels of DA. After carrying out its synaptic function, DA is taken up again into the cytosol by presynaptic cells through the actions of either high-affinity DA transporters (DAT) or low-affinity plasma membrane monoamine transporters. Once in the synaptic neuron, amphetamine exercises a reverse influence on the action of DA transporters (DAT) and forces DA molecules out of storage vesicles and into the synaptic gap [6]. The DA transporter is a sodium-coupled symporter protein responsible for modulating the concentration of extraneuronal DA in the brain [7]. The DA now in the cytosol is then repackaged into vesicles by the action of vesicular monoamine transport, VMAT_2_ [8].

## 3. Metabolism of DA

**Figure 2:**
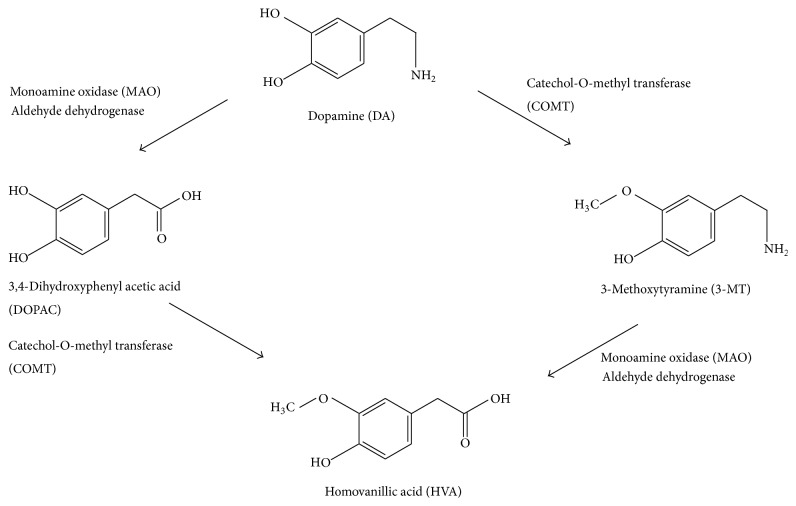
Dopamine metabolism.

**Figure 3:**
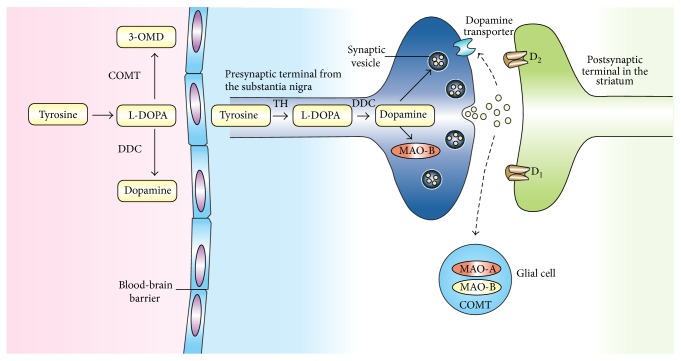
Dopamine metabolism pathways.

The enzymatic breakdown of DA to its inactive metabolites is carried out by catechol-*O*-methyl transferase (COMT) and monoamine oxidase (MAO) (Figure 2). This degradative action can be performed by the MAO isoforms MAO-A and MAO-B. It should be noted that COMT is predominantly expressed by glial cells. In neurons, this enzyme is either missing or found at very low levels. MAO-B is mainly found in astrocytes, whereas MAO-A predominates in catecholaminergic neurons like the cells of the SN. MAO breaks down dopamine to 3,4-dihydroxyphenylacetaldehyde (DOPAL), which in turn is degraded to form 3,4-dihydroxyphenylacetic acid (DOPAC) by the action of the enzyme aldehyde dehydrogenase (Figure 3) [9].

Another pathway for the metabolism of DA involves the enzyme COMT, which converts it to 3-methoxytyramine (3-MT). Then, 3-MT is reduced by MAO to HVA and eliminated in the urine. As a result, the inhibition of monoamine oxidase has been considered as an adjunctive therapy in neurodegenerative disorders such as Alzheimer's and Parkinson's disease (PD) [10]. However, MAO inhibitors are used to increase DA levels and not to decrease hydrogen peroxide production. Actually, neurons have different antioxidant systems, for example, catalase and glutathione, to cope with H_2_O_2_ production. Furthermore, the MAO-derived DOPAC metabolite is probably much more toxic than H_2_O_2_. The inactivation of DA in the brain, striatum, and basal ganglia is mediated by reuptake via DAT followed by enzymatic action of MAO, which breaks it down to DOPAC. Nevertheless, there are few DATs in the frontal cortex, and this leads to the breakdown of DA via another pathway that involves the norepinephrine transporter (NET) on neighboring norepinephrine neurons, proceeded by the enzymatic action of COMT that breaks DA down to 3-MT [11], which may be a way to design therapies against neurological disorders. The velocity of DA degradation is usually faster in the DAT pathway than in NET. In mice, DA is degraded in the caudate nucleus via the DAT pathway within 200 milliseconds, in comparison with 2,000 milliseconds in the frontal cortex [11]. Nondegraded DA is repackaged by VMAT_2_ in the vesicles for reuse.

Dopaminergic neurons are found principally in the VTA of the midbrain, the substantia nigra pars compact, and the arcuate nucleus of hypothalamus. The axons of these neurons project to different areas of the brain through major pathways known as mesocortical, mesolimbic, and nigrostriatal pathways [12]. The mesolimbic pathway connects the VTA to the nucleus accumbens. The somata of the neurons originate in the VTA, and, from there, DA is transported to the nucleus accumbens through the amygdala and the hippocampus. The nigrostriatal pathway joins the substantia nigra with the neostriatum. The neuronal somata are located in the substantia nigra, and the axons of these neurons are ramified into the caudate nucleus and putamen. This pathway is also connected to the basal ganglia motor loop. All of the innervations originating from these pathways explain many of the effects produced when the DA system is activated [13]. For instance, the VTA and the nucleus accumbens connected through the mesolimbic pathway are central to the brain reward system [14].

The modulation of extracellular DA levels occurs by two mechanisms, designated as tonic and phasic DA transmission. The former takes place when a small amount of DA is discharged independent of neuronal activity. This type of discharge is usually regulated by the activity of neurons and neurotransmitter reuptake [15]. The latter occurs when DA is released by the activity of DA-containing cells. Schultz et al. in a study carried out in monkeys reported that this activity is characterized by the irregular pacemaking activity of single spikes and rapid bursts of typically 2–6 spikes in quick succession [16], while Brozoski et al. affirmed that concentrated bursts of activity result in a greater increase of extracellular DA levels than would be expected from the same number of spikes distributed over a longer period of time [17], as a consequence of dopamine metabolism.

## 4. The Reuptake

DA reuptake can be inhibited by cocaine and amphetamines, but each has a different mechanism of action [18]. Cocaine is a DA transporter and norepinephrine transporter blocker. It inhibits the uptake of DA, which results in an increase in DA lifetime, thereby producing an overabundance. Disruptions in these mechanisms following chronic cocaine use contribute to addiction, due, in part, to the unique architecture of the mesocortical pathway. By blocking dopamine reuptake in the cortex, cocaine elevates dopamine signaling at extrasynaptic receptors, prolonging D_1_-receptor activation and the subsequent activation of intracellular signaling cascades, and thus induces long-lasting maladaptive plasticity [19]. Although Barr et al. have identified a novel mechanism by which cocaine promotes activation of D_1_-expressing nAcc neurons, the enhancement of inositol 1,4,5-trisphosphate receptors (IP_3_R) mediated responses via *σ*_1_R activation at the endoplasmic reticulum, resulting in augmented Ca^2+^ release and amplified depolarization due to subsequent stimulation of transient receptor potential canonical channels (TRPC) [20].

## 5. Role of Dopamine in Oxidative Stress

It is well known that mitochondrial dysfunction and oxidative stress contribute in a significant way to the development of PD [21].

A loss of 5–10% of dopaminergic neurons has been found in every decade of aging and an increase in brain oxidative damage is associated with age, and aging is considered a risk factor for PD. The expansive nature of oxidative damage includes mitochondrial dysfunction, DA autooxidation, *α*-synuclein aggregation, glial cell activation, alterations in calcium signaling, and excess-free iron. An increased incidence of PD may be correlated with alterations in the transcriptional activity of various pathways, including nuclear factor erythroid 2-related factor 2, glycogen synthase kinase 3*β*, mitogen activated protein kinase, nuclear factor kappa B, and the reduced activity of superoxide dismutase, catalase, and glutathione with aging [22]. PD is a neurodegenerative disease that usually affects people older than 65 years [23].

Blast-wave-induced traumatic brain injury results in increased hypothalamic expression of oxidative stress markers and activation of the sympathoadrenal medullary axis, due to increased sympathetic excitation. This mechanism may involve elevated AT1 receptor expression and NADPH oxidase levels in the hypothalamus, which is related to DA [24].

The pathway to mitochondrial dysfunction begins with oxidative phosphorylation, which produces superoxide radicals, formed by one superoxide anion, one hydroxyl radical, and free radicals (FR) that come from organic compounds. Alcoxyl, peroxyl, hydrogen peroxide, and singlet oxygen [25], are byproducts that are deposited in the mitochondria, thereby making this organelle the main site for the generation of reactive oxygen species (ROS) within the cell and the first line of defense against oxidative stress [26]. However, superoxide also functions as a signaling molecule, different from signals mediated by hydrogen peroxide, hydroxyl radicals, or peroxynitrite. Although, a well-known role of superoxide is a precursor of reactive hydroxyl radicals by the superoxide-dependent Fenton reaction, the formation of peroxynitrite results in damage to target molecules and leads to pathological disorders, as was reported by Afanas'ev [27]. This author suggested that superoxide signaling depends on nucleophilic reactions. It is necessary to clarify that an oxidant is an element or compound in an oxidation-reduction (redox) reaction that accepts an electron from another species. Due to the fact that it gains electrons, a superoxidant is often a molecule that contains many oxygen atoms and offers a high oxidant capacity.

Studies have suggested that mitochondrial c-Jun N-terminal kinase (JNK) plays a role in the etiology of 6-hydroxydopamine- (6-OHDA-) induced oxidative stress [28]. These authors suggest that 6-OHDA induced cell death through activating PI3K/Akt pathway and inhibiting JNK pathway. On this basis, it was suggested that inhibitors that block the association of JNKs within the mitochondria might be useful neuroprotective agents for the treatment of PD [27], and probably dysfunction in the projections of dopaminergic neurons of the nigrostriatal DA pathway from the substantia nigra to the dorsal striatum would slowly lead to PD [29].

Oxidative stress and hydrogen peroxide (H_2_O_2_) have been implicated as the underlying factors in the initiation and progression of PD. Increases in endogenous H_2_O_2_ in the dorsal striatum attenuated electrically evoked DA release and also decreased basal DA levels [30]. The degeneration of the nigrostriatal pathway in PD is associated with oxidative stress and oxidized DA [31]. On the other hand, selenium transport protein (selenoprotein P) and Sepp1 expressed by neurons of the substantia nigra of the midbrain indicate a role for Sepp1 in the nigrostriatal pathway, which suggests that local release of Sepp1 in the striatum may be important for signaling and/or synthesis of other selenoproteins with neuroprotective activity [32]. Selenoprotein P (Sepp1) and its receptor, apolipoprotein E receptor 2 (apoER2), account for brain retaining selenium better than other tissues, Sepp1-apoER2 interactions supply selenium for maintenance of brain neurons, to protect the severe neurodegeneration and death in mild selenium deficiency [33].

Pharmacological inhibition of brain inflammation and endoplasmic reticulum stress prevented glucose intolerance due to A*β* oligomers (A*β*Os), which act via a central route to affect peripheral glucose homeostasis [34]. A*β* oligomers affect the hypothalamus and reveal a link between hypothalamic dysfunctions in metabolic disorders [35]. The consumption of *β*-phenethylamine- (*β*-PEA-) containing food for a long time is a neurological risk with many pathological consequences [36]. *β*-PEA toxicity is associated with hydroxyl radical (HO) production and oxidative stress generation in dopaminergic areas of the brain. *β*-PEA toxicity may be blocked by inhibition of mitochondrial complex-I [37].

PD has a multifactorial mechanism. Oxidative stress and neuroinflammation, including activation of NADPH-dependent oxidases, play a major role in the progression of dopaminergic cell death [38]. A possible role for DNA repair systems in ageing and neurodegenerative diseases after DNA damage was observed in the brain of individuals affected by neurodegenerative diseases. A study of DNA repair gene polymorphisms (XRCC1 Arg399Gln, XRCC3 Thr241Met XPD Lys751Gln, XPG Asp1104His, APE1 Asp148Glu, and HOGG1 Ser326Cys) suggested that APE1, XRCC1, and XRCC3 genetic variants might be a risk factor for PD by increasing oxidative stress, which might cause the loss of dopaminergic cells in the substantia nigra and locus coeruleus, which could in turn lead to abnormal signal transmission and the development of PD [39].

NADPH oxidase (NOX) was originally identified in immune cells, playing an important microbicidal role. In neurodegenerative and cerebrovascular diseases, inflammation is increasingly being recognized as contributing negatively to neurological outcome, with NADPH oxidase as an important source of superoxide. The activated enzyme complex transports electrons to oxygen, thus producing the superoxide anion (O_2_^∙−^), a precursor of reactive oxygen species, and is the advantage of a targeted NADPH oxidase inhibitor that would inhibit the production of superoxide [40]. Indeed, Nox1/Rac1 could serve as a potential therapeutic target for PD because dopaminergic neurons are equipped with a Nox1/Rac1 superoxide-generating system; however, stress-induced Nox1/Rac1 activation causes oxidative DNA damage and neurodegeneration [41].

Another possible etiology of PD could be due to the loss of serum response factor (SRF), which leads to a decrease in the levels of antiapoptotic proteins, brain-derived neurotrophic factor (BDNF), and Bcl-2, all of which are considered to be a key cause of increased sensitivity to oxidative stress and dysfunction of the SRF-activating mitogen-associated kinase pathway [42]. Organs with a reduced capacity for regeneration like the brain are highly affected by inflammation, and neuroinflammation is recognized as a major contributor to epileptogenesis [43].

Peripheral inflammation provokes brain immune response involving microglial activation, elaboration of proinflammatory cytokines, and reactive oxygen species. Thus, inflammation produces a secondary injury to neurons. A significant part of this response in the brain is mediated by cyclooxygenase (COX) and COX-2 through downstream proinflammatory prostaglandin (PG) signaling [44]. The anti-inflammatory effect of COX in the brain is mediated by PGE_2_ EP_4_ signaling, and the findings of Shi et al. [45] identify the PGE(2) EP3 receptor as a novel proinflammatory, proamyloidogenic, and synaptotoxic signaling pathway. Furthermore, the authors suggest a role of COX-PGE(2) EP3 signaling in the development of AD. These data suggest that LPS induced proinflammatory gene expression in the hippocampus and isolated adult microglia is decreased by a EP_4_ selective agonist. EP_4_ agonists significantly reduced levels of proinflammatory cytokines and chemokines in plasma, indicating that the activation of peripheral EP_4_ gives protection to the brain against systemic inflammation. This suggests that an attractive strategy to prevent the onset and/or delay the progression of neurodegenerative diseases should address the mechanism that is directly implicated in controlling oxidative stress and the inflammatory response. This hypothesis is supported by the work of Kato et al., who proposed that microglial modulation may be a key target in the treatment of various psychiatric disorders [46].

The central nervous system and dopaminergic neurotransmission are associated with the development of addiction. This assertion is supported by the argument that drugs such as nicotine, cocaine, and amphetamine directly or indirectly increase the mesolimbic DA reward pathway and by the neurobiological theory that the DA pathway is pathologically altered in addicted persons [47]. Cocaine, nicotine, and amphetamine have both direct and downstream effects on dopaminergic systems. Cocaine affects the HPA axis and brain nuclei responsible for movements. Cocaine's rewarding effects are through its action on dopaminergic signaling pathways. Therefore, any therapeutic strategy for the abuse of these drugs should target the improvement of the efficacy and tolerability of DA transporters and other molecular targets (Table 1) in clinical disorders.

## 6. The Endocrine System and Dopamine

The depletion of DA may lead to upregulation of the renin-angiotensin system (RAS) to compensate for DA depletion [48]. Nevertheless, hyperactivation of the RAS has many consequences, among which are the aggravation of NADPH oxidase activity and exacerbation of oxidative stress and the microglial inflammatory response and dopaminergic neuron loss [49].

DA is the primary neuroendocrine inhibitor of prolactin secretion by the anterior pituitary gland [50]. The pathway to this inhibitory action begins in the hypothalamic arcuate nucleus, whose neurons produce DA, which is emptied into hypothalamohypophyseal blood vessels of the median eminence, responsible for supplying blood to the anterior pituitary gland, the location of lactotrope cells. These cells secrete prolactin continuously in the absence of DA. Thus, DA is sometimes referred to as the prolactin-inhibiting factor (PIF), prolactin-inhibiting hormone (PIH), or prolactostatin [51].

Wang et al. discovered that D_1_ and D_4_ receptors are responsible for the cognitive-enhancing effects of DA, whereas D_2_ receptors are more specific for motor actions [52]. In humans, antipsychotic drugs that have been found to reduce the activities of DA lead to impairments in concentration and reductions in motivation and inability to experience pleasure (anhedonia) [53]. The prolonged use of DA has been associated with tardive dyskinesia, an irreversible movement disorder [54]. Gonadal hormones are greatly affected by antipsychotic drugs. In women, these drugs are associated with low levels of estradiol and progesterone, while, in men, they significantly reduce the levels of testosterone and dehydroepiandrosterone (DHEA) [55].

The gynecological effects of antipsychotic drugs in women center on hyperprolactinemia, whose main consequences are amenorrhea, cessation of the normal ovarian cycle, loss of libido, occasional hirsutism, false positive pregnancy tests, and the long-term risk of osteoporosis [56]. In men, hyperprolactinemia produced by antipsychotics causes gynecomastia, lactation, impotence, loss of libido, and hypospermatogenesis [57]. Other effects of these drugs include weight gain, drooling, diabetes, sexual dysfunction, dysphoria (abnormal depression and discontent), fatigue, heart rhythm problems, stroke, and heart attack [56].

## 7. Neuroprotective Substances That Alter Dopamine Metabolism

Several studies have reported that antioxidants play an important role in Parkinson's disease [58], and the administration of antioxidant drugs might be used to prevent neuronal death produced by oxidative mechanisms in dopamine metabolism (Table 2).

## 8. Dopamine Metabolism and Antidepressants

Many drugs with antidepressant and antipsychotic properties, including drugs of abuse and endogenous chemicals such as DA, are primarily metabolized in the liver by cytochrome P450 (CYPs) enzymes. Moreover, this degradation can also occur in extrahepatic organs and the brain. Knowledge of brain CYP-mediated metabolism may help in understanding why patients respond differently to drugs used in psychiatry and may predict the risk for psychiatric disorders, including neurodegenerative diseases and substance abuse [59].

Wood reported the role of opioid and cannabinoid transmission in the modulation of food palatability and pleasure of food consumption and noted that this pathway is independent of brain DA [60]. This may explain why food motivation in animals is independent of brain DA concentration. Nevertheless, other consummatory pleasures as feeling or motivating to a person may be more associated with DA.

The brain reward system is strongly associated with DA, which functions to provoke feelings of enjoyment and reinforcement, both of which motivate a person to perform certain works. The release of DA in areas such as the nucleus accumbens and the prefrontal cortex is principally due to rewarding experiences such as food, sex, drugs, and neutral stimuli that are associated with them [61]. Behavioral activation and effort-related processes are regulated by DA of the mesolimbic area, a critical component of brain circuitry.

The principal source of DA in the brain is the dopaminergic neurons of the midbrain. DA is involved in the control of movement and in error signals for reward prediction, motivation, and cognition [61].

Schizophrenia, autism, attention deficit hyperactivity disorders, and drug abuse are other pathological disorders that have been associated with DA dysfunction.

The firing of dopaminergic neurons has been hypothesized to be motivational as a consequence of reward anticipation. The basis of this hypothesis hinges on the fact that a greater reward than expected leads to an increase in the firing of dopaminergic neurons, which consequently increases desire or motivation towards the reward [61]. Nevertheless, recent findings have revealed that some dopaminergic neurons react in consonance with the expectations of reward neurons, while others seem to respond to unpredictability. Moreover, the same findings showed a predominance of reward neurons in the ventromedial region of the substantia nigra pars compact and in the ventral tegmental area. Neurons in these areas project mainly to the ventral striatum and thus might transmit value-related information in regard to reward values [62]. Nonreward neurons are predominant in the dorsolateral area of the substantia nigra pars compacta, which projects to the dorsal striatum and may relate to orienting behavior. Ideas on the role of DA in desire, motivation, and pleasure emanated from studies carried out in animals. In one such study, rats were subjected to depletion of the neostriatum by 99% using 6-hydroxydopamine and nucleus accumbens DA. Foraging behavior is modulated by DA through the activation of brain systems that register reward upon finding a food source [63]. Highly palatable food raises DA levels in monkey, but a prolonged presence of this palatable food makes DA levels decline [64].

DA in the mesolimbic pathway increases general arousal and goal directed behaviors and decreases latent inhibition. These effects augment the creative drive to generate ideas. Thus, creativity is a three-factor model in which the frontal lobes, the temporal lobes, and the mesolimbic DA system [65] play a part. Some authors suggest that the frontal cortex and striatum are more sensitive to oxidative burden, which could be related to the parallel monoamine perturbations [66].

## 9. Consideration for Treatments

Individuals suffering from schizophrenia display an increase in the activity of the dopaminergic system in the mesolimbic pathway. There is decreased activity in the mesocortical pathway. Therefore, these two pathways are blamed for the different sets of symptoms in schizophrenia.

Antipsychotic drugs act as DA antagonists [67]. Psychosis and schizophrenia produce highly abnormal dopaminergic transmission. Nevertheless, clinical studies associating schizophrenia with brain DA metabolism have produced controversial or negative results [68]. The levels of HVA in the cerebrospinal fluid are the same in schizophrenics and controls [69]. Antipsychotic drugs have an inhibitory effect on DA at the level of the receptors and block the neurochemical effects in a dose-dependent manner. Typical antipsychotics commonly act on D_2_ receptors while they atypically act on D_2_ and D_1_, D_3_ and D_4_ receptors, with a low affinity for DA receptors in general [70].

Levodopa is a DA precursor used in various forms to treat PD and dopa-responsive dystonia. Other inhibitors that can be coadministered with levodopa use an alternative metabolic route for producing DA involving catechol-O-methyl transferase. However, oxidative stress and mitochondrial dysfunction can be produced by an increase in endogenous 6-OHDA [71].

As a theoretical possibility, an increase in endogenous 6-OHDA would trigger the formation of Lewy bodies in dopaminergic neurons and eventually lead to their degeneration. Such neurodegeneration could be attenuated using potent antioxidants together with L-DOPA. This would ultimately delay the progression of PD [72]. L-DOPA binds to GPE (Gly-Pro-Glu) by the N-terminal tripeptide of insulin-like growth factor-I. This bond is naturally cleaved in the plasma and brain. GPE has neuroprotective effects since it crosses the blood-CSF and the functional CSF-brain barriers and binds to glial cells, and this tripeptide might represent a promising strategy to supply L-DOPA to Parkinson's patients [73].

The effects of DA on immune cells depend on their physiological state. DA can activate resting T cells, but it can also inhibit them on being activated [74].

This chapter could provide a novel insight into our understanding of the biological mechanisms of neurological disorders and a potential explanation that showed perspectives associated with DA deficits in common clinical disorders that have remained in humans through evolution.

### 9.1. Amphetamines to Treat DA Disorders

Amphetamine acts to increase DA concentration in the synaptic gap through a mechanism that is different from that of cocaine. The structures of amphetamine and methamphetamine are similar to those of DA [75].

Both have two pathways of entrance into the presynaptic terminal bouton, direct diffusion through the neuronal membrane or uptake via DA transporters [76]. The main target of many drugs, such as psychostimulants, nootropics, antidepressants, and some recreational drugs including cocaine, is the DAT. Some stimulants increase the concentration of DA in the presynaptic cleft, an increase that gives rise to an excitatory effect when these drugs are consumed [77].

By increasing the action of the direct pathway in the basal ganglia, DA reduces the effect of the indirect pathway. Macchi et al. found that insufficient DA biosynthesis in dopaminergic neurons causes PD, a condition in which one loses the ability to execute smooth, controlled movements [78].

In addition to the above functions, DA also plays an important role in the neurocognitive function of the frontal lobe by controlling the flow of information from the brain. Hence, DA disorders in this region of the brain can cause a decline in neurocognitive functions, especially in memory, attention, and problem-solving. Moreover, decreased concentrations of DA in the prefrontal cortex are thought to contribute to attention deficit disorder [79].

## 10. Expert Commentary

Disorders such as schizophrenia and PD are associated with altered immune function and changes in brain DA receptors and DA signaling pathways. L-DOPA, DA agonists, inhibitors of DA metabolism, or brain grafts with cells expressing a high level of TH are possible treatment methods for PD because of their ability to correct or bypass an enzymatic deficiency that is the key characteristic of this disease. Another promising target in PD treatment is PPAR-*γ*, which is a key regulator of the immune response. Treatment can also be achieved using agonists with the potential to impact pro- and anti-inflammatory cytokine expression in immune cells at the transcriptional level [80]. Intrastriatal expression of DA synthesizing enzymes could be a promising approach to gene therapy. Expression could be achieved using adenoassociated virus vectors/marrow stromal cells (MSCs) or nonviral intravenous agents involving rat transferrin receptor monoclonal antibodies (TfRmAb) targeted to PE glycated immunoliposomes. The detention or removal of nitrating agents may protect against protein inactivation and limit neuronal injury in PD, thus suggesting the necessity of developing therapeutic agents capable of doing this without interfering with normal neuronal function [81].

The emergence of a highly interesting new area of nonpharmacological treatment of TH dysfunction has occurred in the past few years. TH normalization could provide neuroprotection in PD patients. These new approaches focus on the use of dietetic therapy or the active constituents of plants and phytomedicines, which are believed to provide protection for people suffering from PD [82].

Zhang et al. found that the activation of Akt, a serine/threonine kinase that promotes cell survival and growth, increases the ability of neurons to survive after injury and regenerates lost neuronal connections [83]. These authors suggest that Akt-signaling pathway disinhibition could provide a valuable strategy to enhance survival, function, and integration of grafted DA neurons within the host striatum and improve survival and integration of different forms of neural grafts.

## 11. Five-Year View

In the last few years, the identification of the relationship between immune and neurodegenerative diseases has been demonstrated based on the effect of monoclonal antibodies. Several antibodies that recognize linear A*β* segments also react with fibrils formed from unrelated amyloid sequences. This suggests that reactivity with linear segments of A*β* does not mean that the antibody is sequence specific [84].

In fact, clinical trials on PD have shown that transplants of embryonic mesencephalic DA neurons form new functional connections within the host striatum, but the therapeutic benefits have been highly variable. One obstacle has been poor survival and integration of grafted DA neurons [85].

This mini review indicates that novel therapies may offer significant improvements and target new mechanisms of neurological disorders. These novel therapeutic strategies involve drugs that act not only on the targets of the dopamine transporter but also on other molecular targets to improve drug efficacy and tolerability and obtain the needed improvements in protein homeostasis to alter the metabolism of DA. We recommend that further studies be carried out in different animal and human models.

## Key Points

- Dysfunction of dopamine pathways has been implicated in development of Parkinsonism.
- Common biochemical markers of dopamine are used to monitor its effect and its role in disorders.

## Figures and Tables

**Table 1:** Studies of drugs that alter levels of dopamine or its metabolites in clinical disorders. ↑ up, ↓ down.

Drug	Clinical disorder	Dopamine or metabolites	Ref.
Rasagiline	Antidepressant	MAO-A and MAO-B in the brain ↓	[86]

Methamphetamine (METH)	Addiction	Expression of fosb, fra1, and fra2 in the nucleus accumbens (NAc) ↓	[87]

Ladostigil	Antidepressant	MAO-A and MAO-B in the brain ↓	[86]

Risperidone/donepezil	Parkinsonian features	Dopamine transporter activity ↑	[88]

Cocaine, heroin, or methamphetamine	Addiction	Extracellular dopamine in CNS ↑	[89]

1-Methyl-4-phenyl-1,2,3,6-tetrahydropyridine (MPTP)	Parkinsonian features	Dopamine and TH ↓	[90]

PAOPA	Schizophrenia	Active site of the dopamine D(2) receptor ↓	[91]

Methylphenidate	Cocaine addiction	Dopamine transporter ↓	[92]

Phenelzine	Depression and anxiety disorders	Dopamine levels in brain ↑	[93]

Amphetamine	Attention deficit hyperactivity disorder	Extracellular dopamine ↑	[94]

L-DOPA	Parkinson disease	Brain dopamine levels ↑	[95]

3,4-Methylenedioxymethamphetamine	Addiction	Brain dopamine levels ↑	[96]

Flupenthixol, perphenazine, and zotepine	Tauopathies	Dopamine D(2) receptor ↓	[97]

Asenapine	Acute schizophrenia, manic episodes, bipolar I disorder	Brain dopamine levels ↑	[98]

Pramipexole	Depression	Dopamine receptor D(3) ↑	[99]

**Table 2:** Neuroprotector and antioxidant effect of compounds that alter the dopaminergic metabolism.

Substance	Effects	Tissue or animal models	Ref.
Carnosic acid (CA)	Protection against lipid peroxidation and GSH reduction levels and antiapoptotic and antioxidative action	Human neuroblastoma SH-SY5Y cells	[100]

Hesperidin	Reduction in glutathione peroxidase and catalase activity, total reactive antioxidant potential	Striatum mice	[101]

Carnosic acid	Prevent apoptosis through an increase in glutathione S-transferase P (GSTP) expression via activation of the PI3K/Akt/NF-*κ*B pathway	Human neuroblastoma SH-SY5Y cells	[102]

Alkaloids from *Piper longum* (PLA)	Upregulate the activities of SOD, GSH-Px, CAT, the content of GSH, and the total antioxidant capacity and decrease the content of NOS and the content of MDA, NO	Sustantia nigra and striatum of rats	[103]

Novel (E)-3,4-dihydroxystyryl aralkyl sulfones and sulfoxides	Neuroprotective, antioxidative, and antineuroinflammatory properties	Neuronal cells	[104]

Fenofibrate	Protected against decreased level of DA and excessive production of reactive oxygen species (ROS)	Rats	[105]

2-[[(1,1-Dimethylethyl)oxidoimino]-methyl]-3,5,6-trimethylpyrazine (TBN)	Remarkable neurorescue effects to increase the number of dopaminergic neurons and reduce ROS	Mice and rats	[106]

D-440 is a novel highly selective *D*_3_ agonist	Neuroprotection in cell survival and apoptosis	Dopaminergic MN9D cells	[107]

Gallic acid	Significantly increased passive avoidance memory and total thiol and GPx contents and also decreased MDA levels	Nigral tissue	[108]

Garcinia indica extract	Acted as an effective neuroprotective agent for striatal dopaminergic neurons	Rat	[109]

(±)-*α*-Lipoic acid	Induced reversal of oxidative stress	Human neuroblastoma SH-SY5Y cells	[110]
